# Mangiferin Alleviates Ovalbumin-Induced Allergic Rhinitis via Nrf2/HO-1/NF-κB Signaling Pathways

**DOI:** 10.3390/ijms21103415

**Published:** 2020-05-12

**Authors:** Chun Hua Piao, Yan Jing Fan, Thi Van Nguyen, Chang Ho Song, Ok Hee Chai

**Affiliations:** 1 Department of Anatomy, Jeonbuk National University Medical School, Jeounju, Jeonbuk 54896, Korea; chpiao@jbnu.ac.kr (C.H.P.); fanyj0915@gmail.com (Y.J.F.); vandkh1993@gmail.com (T.V.N.); asch@jbnu.ac.kr (C.H.S.); 2 Institute for Medical Sciences, Jeonbuk National University, Jeounju, Jeonbuk 54896, Korea; 3 Biomedical Research Institute, Jeonbuk National University Hospital, Jeonju, Jeonbuk 54896, Korea

**Keywords:** Mangiferin, allergic rhinitis, inflammation, HO-1/Nrf2 pathway, NF-κB, oxidative stress

## Abstract

Mangiferin (MF), extracted from mango trees, is considered to have anti-inflammatory, anti-apoptotic, and antioxidant effects. However, its effects on allergic rhinitis (AR), remain unclear. We investigated the mechanisms underlying the protective action of MF in ovalbumin (OVA)-induced AR models. AR was induced by OVA challenge in BALB/c mice. Prior to this, MF and dexamethasone were administered. Mice were examined for nasal mucosal inflammation, the generation of allergen-specific cytokine response, and histopathological changes in the nasal mucosa and lung tissue. MF ameliorated nasal symptoms and nasal mucosa inflammation in OVA-induced AR and reduced inflammatory cell infiltration and epithelial disruption in these tissues. MF inhibited the overproduction of Th2/Th17 cytokines and transcription factors. MF downregulated the HO-1/Nrf2 pathways, reduced oxidative stress biomarker levels, and the NF-κB signaling pathways were inhibited. MF exerts protective effects in AR by inhibiting NF-κB and activating HO-1/Nrf2 pathways. MF could be used for the treatment of AR.

## 1. Introduction

Allergic rhinitis (AR) is an IgE-mediated inflammatory disease in the nasal mucosa, characterized by infiltration and activation of inflammatory cells such as mast cells and eosinophils [1]. AR is not a life-threatening disease, but clinical symptoms such as sneezing, rhinorrhea, itching, and stuffy nasal congestion worsen the quality of life and economic burden [1].

The relationship between chronic inflammation and oxidative stress is well documented. Oxidative stress causes an imbalance between the oxidative power and the antioxidant defense system, which is believed to favor oxidative damage implicated in the development of asthma and AR [2,3]. Up until now, researchers have found many natural compounds that have antioxidant and anti-inflammatory activities [4]. 

Mangiferin (1,3,6,7-Tetrahydroxyxanthone C2-β-d-glucoside, MF) is thought to prevent organ damage arising from different causes, and it has anti-inflammatory, anti-apoptotic, and antioxidant effects [5]. In addition, it has been reported to affect the activation or expression of several signaling cascades such as NF-κB, Nrf2/HO-1, mitochondrial-dependent pathways, and to target several cytokines, interleukin (IL)-6, and antioxidant enzymes, such as superoxide dismutase (SOD) and catalase (CAT) [5,6,7,8]. MF has been reported to inhibit NF-κB activation and a series of proinflammatory cytokines [9]. Furthermore, several mechanism studies have shown that MF exhibits pharmacological actions through activation of Nrf2 [5,10]. The interaction between Nrf2 and NF-κB is interesting because numerous phytochemicals that have anti-inflammatory, anti-oxidative, or anti-cancer properties suppress NF-κB signaling and activate the Nrf2 pathway [11].

Therefore, our aim was to study the anti-allergic and antioxidant properties of MF using a mouse model of ovalbumin (OVA)-induced allergic rhinitis (AR) to find out their effects on the activation of NF-κB signaling and Nrf2/HO-1 pathway. In addition, we also assessed whether the regulation of MDA, SOD, and proinflammatory and Th1/Th2/Th17 cytokines by MF accounted for its protective role in AR animal models.

## 2. Results

### 2.1. Effects of MF on Nasal Symptoms of OVA-Induced AR Mice

**Figure 1:**
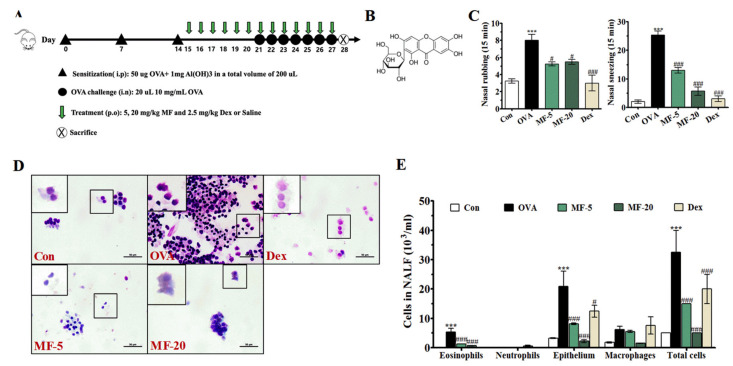
Effect of Mangiferin (MF) on nasal symptoms and allergic inflammation of OVA-induced allergic rhinitis (AR) mice. (**A**) Experimental protocol (**B**) Chemical structure of MF. (**C**) The frequency of nasal rubbing and sneezing numbers after the final OVA challenge was assessed by counting for 15 min without anesthesia. (**D**) Cytospin cell preparations were made by NALF and stained with Diff-Quik; Scale bar= 50 µm. (**E**) Total and differential cell numbers were counted using a hemocytometer. *** p < 0.001, significantly different from the value of the Control group. *^#^ p < 0.05, ^###^ p < 0.001*, significantly different from the value of OVA group.

AR is typically characterized by chronic inflammation, nasal itching, sneezing, and rhinorrhea [12]. Thus, we assessed the effect of MF on nasal symptoms of AR. The frequency of nasal rubbing and sneezing was counted for 15 min after the final OVA challenge. As shown in Figure 1C, an increased frequency of nasal rubbing and sneezing was observed in the OVA group. Notably, mice treated with MF displayed a remarkable dose-dependent inhibitory effect. Dex was used as a positive control and significantly reduced the nasal symptoms. We also found that there was no significant change in the lung W/D weight ratio among all the groups (data not shown). These results indicated that MF plays a therapeutic role in AR.

### 2.2. Effects of MF on the Infiltration of Inflammatory Cells in the NALF of OVA-induced AR Mice

To explore the role of MF on nasal inflammation, we evaluated the leukocyte levels of NALF. The total cell count and the differential counts for eosinophils, neutrophils, and macrophages in NALF were determined. Consistent with the nasal symptoms, the OVA group had a significantly higher number of total cells compared with the Control group, and most of these cells were epithelial cells and a few inflammatory cells such as eosinophils, neutrophils and macrophages were shown. However, these cellular counts in MF and Dex groups were found to be significantly decreased (Figure 1D,E).

### 2.3. Effects of MF on the Histopathological Changes of the Nasal Mucosa of OVA-Induced AR Mice

**Figure 2:**
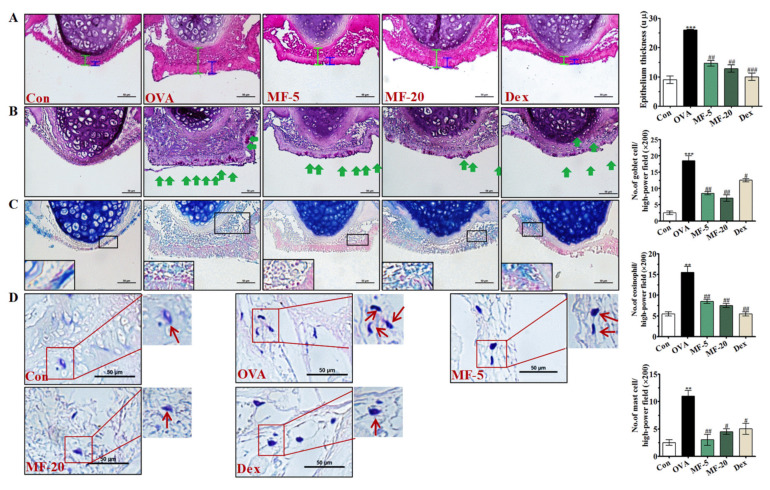
Effect of MF on histological changes in nasal tissues of OVA-induced AR mice. (**A**) The general histology, epithelium thickness of nasal mucosa, HE staining; Scale bar= 50 µm. (**B**) Goblet cells hyperplasia. PAS staining; the green arrow indicates the goblet cell; Scale bar= 50 µm. (**C**) Infiltration of the eosinophils, Giemsa staining; Scale bar= 50 µm. (**D**) Mast cells immigration in the nasal mucosa. Giemsa staining; Scale bar= 50 µm. Red arrow indicates the mast cells. Values are presented as the mean ± SEMs (*n*= 6 per group). ** p < 0.01, *** p < 0.001, significantly different from the value of Control group. *^#^ p < 0.05, ^##^ p < 0.01, ^###^ p < 0.001*, significantly different from the value of OVA group.

Subsequently, we assessed the effects of MF on the histopathological changes in the nasal mucosa by HE staining. Considerable changes of epithelial disruption, mucosal detachment, and inflammatory cell infiltration were observed in the nasal mucosa of OVA-induced AR mice while the nasal mucosa of MF-treated mice was notably protected from being damaged by OVA, which was evaluated by the thickness of the nasal epithelium. In addition, we also analyzed the thickness of epithelial cells in the nasal mucosa and observed that MF treatment also reduced the swelling of epithelial cells caused by OVA in AR mice (Figure 2A). Additionally, the PAS staining results showed that the goblet cell hyperplasia of the OVA group was remarkably upregulated. However, treatment with MF notably downregulated the goblet cell hyperplasia (Figure 2B).

Eosinophils and mast cells are important effector cells in allergic inflammation and also associated with the development of allergic disorders, including AR and asthma [13]. We explored whether MF treatment plays an essential role in eosinophil infiltration. Significantly higher eosinophil infiltration was apparent in the OVA group compared to the Control group. Upon MF administration, eosinophil infiltration reduced significantly compared to the OVA group (Figure 2C). Furthermore, the number of mast cells in the nasal mucosa of mice in each group was evaluated by Giemsa staining. Compared to the Control group, the number of mast cells was shown to be significantly increased in the OVA group. In contrast, MF treatment reduced mast cell infiltration into the nasal cavity, which indicated that MF treatment could reduce the infiltration of mast cells into the nasal mucosa of AR mice (Figure 2D). In addition, the numbers of eosinophils, goblet cells, and mast cells infiltrating the nasal mucosa were estimated quantitatively in the histologic sections of each group as shown in Figure 2. Interestingly, the protective effect of MF-20 on inflammatory cell production was better than that of MF-5.

### 2.4. Effects of MF on the Serum Levels of OVA-Specific IgE, IgG1, and IgG2a of OVA-Induced AR Mice

**Figure 3:**
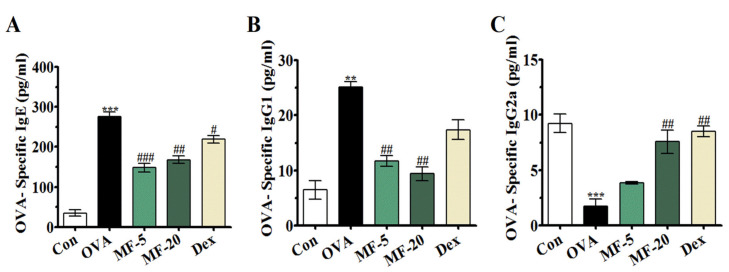
Effect of MF on the serum levels of OVA-specific IgE, IgG1 and IgG2a expressions in OVA-induced AR mice. The serum levels of OVA-specific IgE, IgG_1_, and OVA-specific IgG _2a_. Values are presented as the mean ± SEMs (*n*= 6 per group). ** p < 0.01, *** p < 0.001, significantly different from the value of Control group. *^#^ p < 0.05, ^##^ p < 0.01, ^###^ p < 0.001*, significantly different from the value of OVA group.

To further assess whether MF could ameliorate the inflammatory response generated in AR, we examined the levels of OVA-specific immunoglobulins in serum by ELISA. As shown in Figure 3, the levels of OVA-specific IgE and IgG1 were significantly higher in the OVA group, while the serum level of OVA-specific IgG2a in this AR model was significantly decreased compared with the Control group. Upon MF treatment, the production of OVA-specific IgE and IgG1 in the serum was reduced. However, regarding OVA-specific IgG2a, lower-dose MF treatment exhibited a weak tendency to increase its production, whereas high-dose MF could markedly lower the level of OVA-specific IgG2a compared with that of the OVA group. (Figure 3). These results suggested that MF attenuated allergic symptoms by suppressing the production of OVA-specific IgE and IgG1 while increasing the Th1-related OVA-specific IgG2a.

### 2.5. Effects of MF on Lung Histology and Mast Cell Migration in OVA-Induced AR Mice.

**Figure 4:**
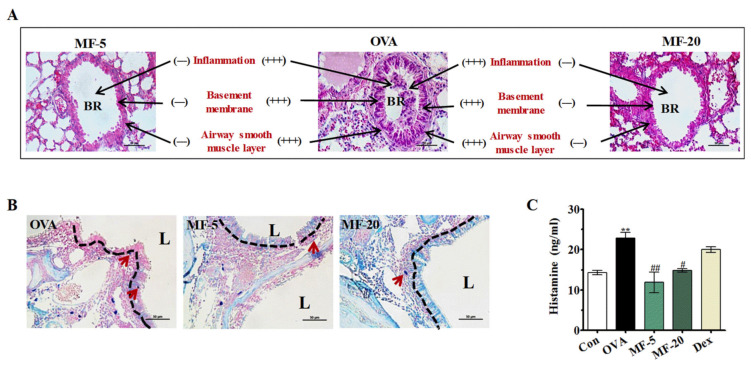
Effect of MF on lung histology changes and mast cell migration in OVA-induced AR mice. (**A**) Lung tissues from each group were stained with HE; Scale bar=100. (**B**) Mast cells immigration and migration to the epithelium. Giemsa staining; Scale bar= 50 µm. (**C**) The serum levels of histamine. Values are presented as the mean ± SEMs (*n*= 6 per group). BR: Bronchus; L: airway lumen. ** p < 0.01, significantly different from the value of Control group. *^#^ p < 0.05, ^##^ p < 0.01,* significantly different from the value of OVA group.

HE staining was used to investigate the effects of MF on lung histological changes. The results showed that OVA-induced AR mice showed significant inflammatory cell infiltration and airway epithelial thickening in the peribronchial and perivascular regions. However, treatment of MF or Dex markedly attenuated OVA-induced pathological changes (Figure 4A). In addition, the migration of mast cells from the epithelium in the MF group was inhibited compared to the OVA group (Figure 4B). Accumulated studies showed that histamine secreted from mast cells plays a major role in the pathogenesis of the allergic reaction to AR [14]. To ascertain the anti-allergic mechanism of MF on OVA-induced AR mice, the levels of histamine were measured in each group of mice. As shown in Figure 4C, we observed significantly increased levels of histamine in the OVA group compared to the Control group. However, such OVA-induced histamine release can be markedly suppressed by MF treatment.

### 2.6. Effects of MF on the Levels of Th1, Th2, Th17 and Proinflammatory Cytokines, and Related Transcription Factors in OVA-induced AR Mice

**Figure 5:**
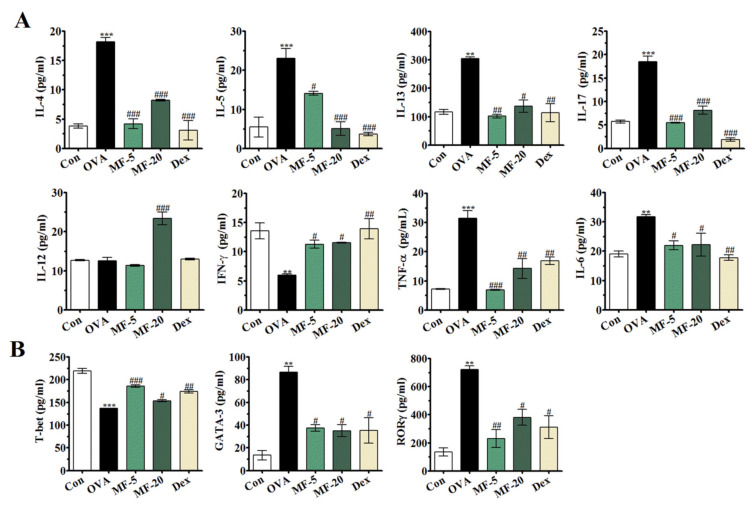
Effect of MF on Th1/Th2/Th17 and pro-inflammatory cytokines and related transcription factor in OVA-induced AR mice. The levels of (**A**), Th2 cytokines, IL-4, IL-5, IL-13; Th17 cytokine, IL-17; Th1 cytokines IL-12, IFN- γ and pro-inflammatory cytokines TNF-α and IL-6 were determined in NALF. (**B**) Analysis of T-bet, GATA-3 and RORγ expression levels in NALF. Values are presented as the mean ± SEMs (*n*= 6 per group). ** p < 0.01, *** *p < 0.001*, significantly different from the value of Control group. *^#^ p < 0.05, ^##^ p < 0.01, ^###^ p < 0.001*, significantly different from the value of OVA group.

Next, we determined whether MF affected the Th1/Th2/Th17 imbalance in AR mice. Production of inflammatory mediators in AR containing IL-4, -5, -12, 13, -17, and IFN-γ in NALF was measured by ELISA. Notably, compared to the Control group, the OVA group was shown to have apparently increased levels of Th2 cytokines IL-4, IL-5 and IL-13, and Th17 cytokine IL-17 in NALF, while the Th1 cytokines IL-12 and IFN-γ were reduced, showing typical Th2 polarization characteristics. Interestingly, therapeutic administration of MF offered remarkable protection against the Th1/Th2/Th17 imbalance by upregulating IL-12 and IFN-γ, and downregulating IL-4, -5, -13, and 17 levels in NALF (Figure 5A). These results highlighted the significantly restrictive effect of MF on the Th1/Th2/Th17 imbalance.

After antigen/IgE stimulation, activated mast cells release allergic mediators, including histamine and proinflammatory cytokines associated with the recruitment of inflammatory cells [1]. To ascertain the anti-allergic mechanism of MF on OVA-induced AR mice, the levels of proinflammatory cytokines IL-6 and TNF-α in NALF were evaluated in each group of mice. As shown in Figure 5A, we observed significantly increased levels of IL-6 and TNF-α in the OVA group compared to the Control group. However, such OVA-induced TNF-α release could be markedly suppressed by MF treatment, although MF only slightly ameliorated IL-6 levels (Figure 5A). These data suggested that MF may improve OVA-induced AR symptoms by blocking the release of proinflammatory cytokines.

The expression of transcription factor T-bet, GATA-3, and RORγ were detected by ELISA. We discovered that the levels of transcription factors, GATA-3 and RORγ, varied drastically in the OVA group compared to the Control group, while T-bet was decreased. In contrast, MF treatment suppressed the production of GATA-3 and RORγ and upregulated T-bet (Figure 5B), providing further confidence in the data.

### 2.7. MF Inhibits OVA-Induced Activation of NF-κB Signaling in NALF

**Figure 6:**
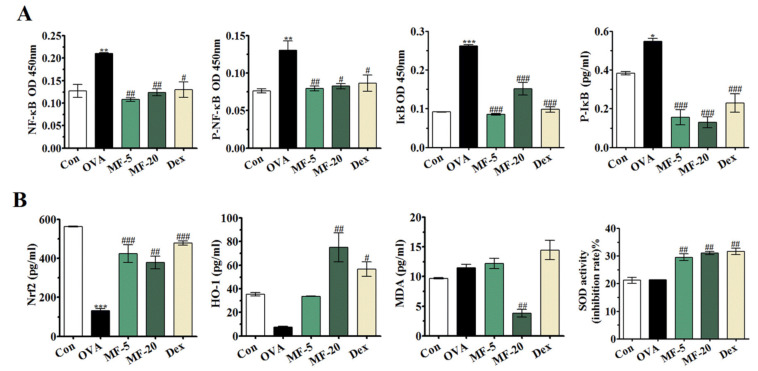
Effect of MF on NF-кB and Nrf2/HO-1 signaling pathways in OVA-induced AR mice. (**A**) Detection of NF-κB, P-NF-κB, IκB and P-IκB in NALF. (**B**) Production of HO-1, Nrf2, SOD, and MDA in NALF. Values are presented as the mean ± SEMs (*n*= 6 per group). * p < 0.05, ** p < 0.01, *** p < 0.001, significantly different from the value of Control group. *^#^ p < 0.05, ^##^ p < 0.01, ^###^ p < 0.001*, significantly different from the value of OVA group.

Translocation of NF-κB is a hallmark of the molecular inflammatory phenomenon involved in the expression and the production of proinflammatory mediators and cytokines [15]. Here, we explored the contribution of NF-κB activation in inflammation using a MF treated AR model. NF-κB activation involves upregulated IκB and NF-κB phosphorylation [16,17]. Compared with the Control group, the expression of NF-κB, p-NF-κB, and IκB in the OVA group markedly increased, whereas MF treated mice exhibited a decrease in NF-κB signaling. Moreover, the expression of NF-κB, p-NF-κB and IκB were decreased under Dex administration (Figure 6A). The results showed that MF inhibited allergic inflammatory responses by inhibiting NF-κB activation.

### 2.8. Effects of MF on Lipid Peroxidation and Antioxidant Enzyme Activity, and Nrf2/HO-1 Signaling Pathway

Lipid peroxidation is a well-known mechanism of cell damage. It produces byproducts, such as lipid peroxides and aldehydes, and finally leads to the destruction of membrane lipids. Malonaldehyde (MDA) represents the final product of this process derived from the decomposition of polyunsaturated fatty acids and related esters. Measurement of MDA provides an accurate and established index of oxidative damage.

In order to determine the mechanism of MF’s antioxidant effect on AR, we aimed to identify the level of MDA in NALF by ELISA. Figure 6B shows that the basal level of lipid peroxidation, measured as the formation of MDA, is markedly increased in OVA-induced AR, indicating the accumulation of oxidative damage by inflammation. MF treatment significantly reduced the concentration of MDA compared to the OVA group, only at the higher concentration of 20 mg/kg. These observations indicate a significant correlation between the basal levels of MF and MDA. 

Furthermore, MF treatment significantly increased the activity of the antioxidant enzyme SOD. MF reduced oxidative stress and enhanced antioxidant enzyme level in OVA-induced AR. These results demonstrated that MF could improve OVA-induced oxidative stress through increased activity of SOD in NALF. The effect of MF on the Nrf2 signaling pathway was detected in NALF. In relation to changes in oxidative stress in AR, the OVA group exhibited significantly downregulated expression of Nrf2 as well as that of its downstream protein HO-1 (Figure 6B). Nevertheless, both selected doses of MF markedly upregulated the Nrf2/HO-1 signaling pathway (Figure 6B). 

## 3. Discussion

MF is a promising antioxidant with tremendous health-related properties such as antiviral, anticancer, anti-diabetic, anti-oxidative, anti-aging, immunomodulatory, hepatoprotective, and analgesic effects [18]. In previous studies, MF has been demonstrated to exert an anti-asthmatic effect by decreasing the level of Th2 cytokines IL-4 and IL-5 in the bronchoalveolar lavage fluid and lymphocyte culture supernatant [19]. In this study, we found for the first time that MF administration could affect immune inflammation and oxidative stress in an OVA-induced AR model. Our results showed that MF treatment could inhibit the production and secretion of OVA-specific immunoglobulins in serum and allergic cytokines like IL-4, IL -5, IL -6, IL-13, IL-17, TNF-α and NF-κB activation in NALF caused by OVA induction. Moreover, MF also decreased the OVA-induced thickness of epithelial cells, eosinophils, goblet cells, and mast cells in the nasal mucosa and allergic symptoms of nasal rubbing and sneezing in AR mice. Furthermore, MF treatment also significantly improved the expressions of HO-1/Nrf2, SOD, and decreased the MDA concentration, suggesting that MF has a potential application to attenuate OVA-induced AR. 

The function of Th2 cells in allergic diseases is to induce the release of allergen-specific IgE Ab by B cells and promote the infiltration of eosinophils and mast cells into target tissues [20]. Allergic responses involve many inflammatory cells, especially the two primary cells that carry out this complex reaction are eosinophils and mast cells [21]. Eosinophil infiltration is a hallmark of AR mucosal inflammation [22]. Mast cells are widely distributed on the mucosal surface and connective tissues, penetrating into the areas of inflammation associated with chronic atopic or allergic diseases [23]. In this study, our results showed that the nasal mucosa structures were rearranged, with progressively decreased infiltration of eosinophils and mast cells, reduced expressions of OVA-specific IgE, IgG1, and attenuation of the nasal symptoms, including nasal rubbing and sneezing. Thus, we demonstrated that MF exerts anti-allergic effects through inhibition of inflammatory cells infiltration by reducing OVA-specific immunoglobulins and nasal symptoms.

The traditional view is that imbalance between Th1 and Th2 leads to development AR [24]. IL-4 increases IgE synthesis in B cells and participates in allergic reactions [25]. IL-5 is a major differentiation and maturation factor for eosinophils, and at the same time, it is important for supporting eosinophil activation, development, survival, and response to other cytokines [26]. IL-13 is released by activated T cells, B cells, and mast cells [27]. In the present study, our results showed that IL-4, IL-5, IL-13 as well as GATA-3 levels in NALF were significantly decreased in all MF-treated groups. In addition, the levels of IL-12 and IFN-γ, Th1 cytokines and its transcription factor T-bet, were higher in MF treatment groups than those in the OVA group. Moreover, we found that MF markedly inhibited IL-17 and its transcription factor RORγ. Therefore, we detected that MF exerts an anti-allergic effect through Th1/Th2/Th17 responses in AR.

Recent studies showed that allergic inflammation is mediated by NF-κB activation [28]. To investigate the anti-inflammatory mechanism of MF, its action on the NF-κB signaling pathway was detected. The results of this study showed that OVA-induced NF-κB activation was significantly inhibited upon MF treatment. Thus, it can be suggested that MF exerts anti-inflammatory action by regulating NF-κB activation.

MF belongs to xanthonoids, one of the several types of natural compounds collectively called polyphenols present in significant levels in higher plants and in different parts of the mango tree, such as the peel, stalks, leaves, barks, kernel, and stone [29]. Due to its unique structure with C-glycosidic linkage, MF has potent antioxidant and free radical scavenging properties [6,7]. In this study, we observed that MF could significantly inhibit OVA-induced oxidative stress, as evidenced by decreased MDA and increased SOD levels. These results suggested that MF protects against OVA-induced AR by inflammatory and oxidative responses. 

Nrf2 is a transcription factor implicated in the transactivation of gene encoding detoxifying enzymes [30]. Nrf2/HO-1 signaling pathways are known to play a critical role in the regulation of oxidative stress [31]. Meanwhile, the antioxidant effect of MF via increased expression of Nrf2 and HO-1 was confirmed, and consistent with the results previously recorded in a hepatotoxic model [32]. Recently, Nrf2 has been reported to be involved in the inflammatory response. In this study, we observed that MF could upregulate the Nrf2 and HO-1 expression levels, indicating that Nrf2/HO-1 may be involved in the antioxidant activity of MF, as mentioned in other studies. To the best of our knowledge, this finding is the first direct evidence of the protective role of MF through the Nrf2/HO-1 signaling pathway in OVA-induced AR.

## 4. Materials and Methods

### 4.1. Chemicals and Reagents

OVA (grade VI), and MF were purchased from Sigma-Aldrich (St, Louis, MO, USA). Aluminum hydroxide adjuvant was purchased from Pierce (Thermo Scientific, Rockford, MD, USA), and dexamethasone (Dex) was purchased from Innovative Research of America (Toledo, OH, USA). Saline (NaCl 0.9%; B. Braun Medical BV, Oss, The Netherlands).

### 4.2. Animals 

Specific pathogen-free male 5-week-old BALB/c mice were purchased from Damool Science (Dae-jeon, Korea). These mice were housed six per cage in a laminar air-conditioned room maintained at 23 ± 2 °C at a relative humidity of 55 ± 10% with a 12 h dark/light cycle throughout the study. All animal experiments were performed in accordance with the Care and Use of Experimental Animals and were approved by the Animal Research Committee of Jeonbuk National University Laboratory Animal Center (CBNU 2019-071).

### 4.3. The OVA-induced AR Model

Animals were divided into five groups, consisting of six mice in each and treated for 28 days as follows:

Group 1: “Control group (Con)”, animals without sensitization and challenge; on days 16 to 28, animals received once-daily oral administration of saline as a negative control.

Group 2: “OVA”, animals were immunized by intraperitoneal injection of 50 μg OVA with 1 mg—Alum in a total volume of 200 μL on days 0, 7 and 14. Next, for five consecutive days from 21 to day 27 after the start of the sensitization period, these mice were challenged with OVA (10 mg/mL, 20 μL/ nostril) via intranasal instillation. From days 15 to 27, animals were also treated once daily with saline by oral administration as a positive control.

Group 3: “Mangiferin treated group (MF-5)”, animals were immunized and challenged following the same protocol as for the “OVA” group. From days 15 to 27, animals were also treated once daily with MF (5 mg/kg) by oral treatment for 13 consecutive days. From days 21 to 27 MF was administered 1 h before every OVA challenge.

Group 4: “Mangiferin treated group (MF-20)”, animals were immunized and challenged as per the above protocol. On days 15 to 27, animals were also treated once daily with MF (20 mg/kg) by oral treatment for 13 consecutive days. From days 21 to 27 MF was administered 1 h before every OVA challenge.

Group 5: “Dexamethasone treated group (Dex)”, animals were immunized and challenged using the same protocol as for other groups. From days 15 to 27, animals were also treated once daily with Dex (2.5 mg/kg) by oral treatment for 13 consecutive days. From days 21 to 27 Dex was administered 1 h before every OVA challenge.

Animals were sacrificed at 24 h on day 28, and nasal and lung tissues along with nasal lavage fluid (NALF) were obtained for analysis (Figure 1A).

The concentration of Mangiferin was used in this study according to the method described by Mahmoud-Awny et al. [33] and Wang et al. [10] with some modifications.

### 4.4. Lung Wet/Dry Weight Ratio

Lung edema was assessed by measuring the tissue wet/dry (W/D) weight ratio. The right lung was excised, and the wet weight recorded. The lungs were placed in an incubator at 80 °C for 24 h, and then the W/D weight was calculated.

### 4.5. Nasal Symptom Scores

The frequency of nasal rubs and sneezing behaviors were counted to assess nasal symptoms. After the final OVA intranasal challenge from days 21 to 27, the numbers of nasal rubbing and sneezing behaviors were recorded for a 15 min period, and experimental conditions were counted by blinded observers. 

### 4.6. Collection and Measurement of Serum Levels of OVA-Specific Immunoglobulins

Twenty-four hours after the final OVA challenge, blood specimens were collected from retro- orbital plexus, and one drop was smeared on a slide and stained with Diff-Quik staining (Sysmex Co., Kobe, Japan). Serum was obtained by centrifugation at 1000 g for 10 min at 4 °C (Centrifuge 5403; Eppendorf, Hamburg, Germany), and then serum was stored immediately at −80 °C for future analysis. The serum levels of OVA-specific IgE, OVA-specific IgG_1,_ and OVA-specific IgG_2_ were measured using enzyme-linked immunosorbent assay (ELISA), and the optical density was measured at 450 nm in accordance with the protocol provided by the manufacturer.

### 4.7. Collection and Analysis of NALF

Twenty-four hours after the last OVA challenge, NALF was collected by cannulating the upper part of the trachea in the nasal cavity direction and washing with 1 mL saline. Supernatants were collected for measurement of cytokines and stored at −80 °C until used. The total cell numbers in NALF were determined by trypan blue exclusion using a hemocytometer. Subsequently, an aliquot of 150–200 µL was centrifuged in a cytospin cytocentrifuge (Centrifuge 5403; Eppendorf, Hamburg, Germany). The differential cell counts of eosinophils, neutrophils, and lymphocytes were examined from the centrifuged preparations that were followed by Diff-Quik staining (Sysmex Co., Kobe, Japan) by counting 100 or more cells from each sample at 200 × magnification.

### 4.8. Histopathological Evaluation of Nasal Mucosa and Lung Tissues

After analyzing the NALF, the head of the mouse was removed, fixed in 10% paraformaldehyde for 3 days, then decalcified for 7 days in ethylenediamine triacetic acid, and thereafter, embedded in paraffin wax. Moreover, the lung lobes were removed for histological examination; lungs were fixed in 10% paraformaldehyde for 3 days; nasal and lung tissues were sectioned coronally into 5-µm slices for histological assessment. Subsequently, the number of inflammatory cells was assessed with a randomly selected high-power field at 400 × magnification.

Hematoxylin and eosin (HE) staining was used to assess the general morphological structure of nasal mucosa and lung tissue.

PAS staining was performed on the nasal septum mucosa sections to visualize the development of goblet cell hyperplasia.

Giemsa staining was used for visualization of mast cell and eosinophil infiltrations in the nasal mucosa. Eosinophils were defined morphologically by the presence of granules in the cytoplasm and bilobal nucleus and were counted under the microscope. 

### 4.9. Assay of Lipid Peroxidation, Antioxidant Enzyme Activities, and Nrf2/HO-1 and NF-κB Signaling Pathways

Supernatants were tested for lipid peroxidation by collecting and measuring the concentration of malondialdehyde (MDA). The activity of Superoxide dismutase (SOD) was analyzed according to Misra and Fridovich. In the next 4 min, adrenochrome formation was recorded at 475 nm in accordance with the protocol provided by the manufacturer. The levels of Nrf2 and HO-1 in NALF were assayed by ELISA kits (R&D Systems, Minneapolis, MN, USA), following the manufacturer’s instructions. Moreover, the supernatants were obtained for analysis of NF-кB, IкB, P-IкB, and P-NF-кB in NALF. 

### 4.10. Measurement of Th1/Th2/Th17 and Proinflammatory Cytokines and Related Transcription Factor Levels

In the Th1 response, the levels of anti-inflammatory cytokines (Th1-associated cytokines) such as IFN-γ and IL-12 levels, and relative transcription factor T-bet in NALF were assayed by ELISA kits (R&D Systems), following the manufacturer’s instructions.

In the Th2 response, we examined the secretion of the inflammatory cytokines (Th2-associated cytokines) IL-4, IL-5, and IL-13, and relative transcription factor GATA-3 in NALF by ELISA, following the manufacturer’s instructions.

In the Th17 response, Th17-associated cytokine, IL-17, and relative transcription factor RORγ in NALF were measured, following the manufacturer’s instructions.

In the proinflammatory cytokine response, the levels of IL-6 and TNF- α in NALF were measured, following the manufacturer’s instructions.

### 4.11. Statistical Analysis

Results are analyzed using Graph Pad Prism software (v5.0, La Jolla, CA, USA) and expressed as mean ± SEMs over the number of experiments. Statistical analysis was adjusted to a Student’s t-test and ANOVA with Dunnett’s test. Statistical significance was defined at the 95% confidence level (*p* < 0.05).

## 5. Conclusions

In summary, our study revealed that MF exerts an anti-allergic, anti-inflammatory, and antioxidant effect by regulation of Th1/Th2/Th17 cytokines and related transcription factors, proinflammatory cytokines, and oxidative stress in AR mice. MF attenuated OVA-induced allergic nasal inflammation by activating the Nrf2/HO-1 signaling pathway and blocking the activity of NF-κB. These results suggest that MF may be recommended as a potential treatment for AR. The results of this study demonstrated that MF exerts anti-inflammatory and antioxidant effects, particularly on nasal tissues in a murine model of AR. Meanwhile, these results provide underlying mechanistic evidence for the clinical use of MF to treat AR.
